# Supplementary material for: Prevalence of bacterial vaginosis and aerobic vaginitis and their associated risk factors among pregnant women from northern Ethiopia: A cross-sectional study
Source: PLoS One. 2022 Feb 25;17(2):e0262692. doi: 10.1371/journal.pone.0262692 (PMC8880645; doi:10.1371/journal.pone.0262692)
Supplement: S1 File — (PDF) [file pone.0262692.s001.pdf]

Research plan questionnaire

Name and last name:

Date of recruitment:

Phone/ Mobile number:

Address:

Questions		Answer
Birth date		
Residence	Urban	
	Rural	
Educational status	Unable to write and read	
	Primary school	
	Secondary school	
	College and above	
Occupational status	Employee	
	Housewife	
	Others	
Marital status	Unmarried	
	Married	
	Divorced/widowed	
HIV	Positive	
	Negative	
Condom use	Yes	
	No	
Fungal infection	Yes	
	No	
Number of LTSP	One	
	Two and above	

Number of pantyliner used per day	1-2/day	
	1/2-4 days	
Douching using water	Once daily	
	More than one per day	
Douching using soap	Yes	
	No douching	
Previous BV/GTI	Yes	
	No	
History of abortion	Spontaneously	
	Induced	
	No	
Gestational age	First trimester	
	Second trimester	
	Third trimester	
Number of pregnancy	Primigravida	
	Multigravida	
BV score	Normal	
	Intermediate	
	BV	
AV score	Normal	
	Light	
	Moderate	
	Severe	
