# Supplementary material for: Prevalence of bacterial vaginosis and aerobic vaginitis and their associated risk factors among pregnant women from northern Ethiopia: A cross-sectional study
Source: PLoS One. 2022 Feb 25;17(2):e0262692. doi: 10.1371/journal.pone.0262692 (PMC8880645; doi:10.1371/journal.pone.0262692)
Supplement: S1 Table — (DOCX) [file pone.0262692.s002.docx]

**Supplementary Information**

Table 1. Socio-demographic characteristics of pregnant women at Ayder comprehensive Specialized Hospital from February to June 2019.

| **Variables** | | **Count** | **Percent** |
| --- | --- | --- | --- |
| Age | ≤ 20 years | 37 | 8.7 |
|  | 21-29 years | 270 | 64.0 |
|  | ≥ 30 years | 115 | 27.3 |
| Residence | Urban | 400 | 94.8 |
|  | Rural | 22 | 5.2 |
| Educational status | Unable to write and read | 19 | 4.5 |
|  | Primary school | 82 | 19.4 |
|  | Secondary school | 171 | 40.5 |
|  | College and above | 150 | 35.5 |
| Occupational status | Employee | 111 | 26.3 |
|  | Housewife | 223 | 52.8 |
|  | Others | 88 | 20.9 |
| Marital status | Unmarried | 15 | 3.6 |
|  | Married | 402 | 95.3 |
|  | Divorced/widowed | 5 | 1.1 |
| HIV | Positive | 12 | 2.8 |
|  | Negative | 410 | 97.2 |
| Condom use | Yes | 31 | 7.3 |
|  | No | 391 | 92.7 |
| Fungal infection | Yes | 33 | 7.8 |
|  | No | 389 | 92.2 |
| Number of LTSP | One | 363 | 86.0 |
|  | Two and above | 59 | 14.0 |
| Number of pantyliner used per day | 1-2/day | 298 | 70.6 |
|  | 1/2-4 days | 124 | 29.4 |
| Douching using water | Once daily | 119 | 28.2 |
|  | More than one per day | 303 | 71.8 |
| Douching using soap | Yes | 43 | 10.2 |
|  | No douching | 379 | 89.8 |
| Previous BV/GTI | Yes | 62 | 14.7 |
|  | No | 360 | 85.3 |
| History of abortion | Spontaneously | 64 | 15.2 |
|  | Induced | 32 | 7.6 |
|  | No | 326 | 77.3 |
| Gestational age | First trimester | 51 | 12.1 |
|  | Second trimester | 207 | 49.1 |
|  | Third trimester | 164 | 38.9 |
| Number of pregnancy | Primigravida | 158 | 37.4 |
|  | Multigravida | 264 | 62.6 |
| BV score | Normal | 263 | 62.3 |
|  | Intermediate | 74 | 17.5 |
|  | BV | 85 | 20.1 |
| AV score | Normal | 388 | 91.9 |
|  | Light | 19 | 4.5 |
|  | Moderate | 4 | 0.9 |
|  | Severe | 11 | 2.6 |

HIV = Human immunodeficiency virus, LTSP = Lifetime sexual partner, AV = Aerobic vaginitis, BV = Bacterial vaginosis, GTI = Genital tract infection
