# Supplementary material for: Prevalence of bacterial vaginosis and aerobic vaginitis and their associated risk factors among pregnant women from northern Ethiopia: A cross-sectional study
Source: PLoS One. 2022 Feb 25;17(2):e0262692. doi: 10.1371/journal.pone.0262692 (PMC8880645; doi:10.1371/journal.pone.0262692)
Supplement: S2 Table — (DOCX) [file pone.0262692.s003.docx]

**Supplementary Information**

Table 2. Prevalence of vaginal infections among pregnant women at Ayder Comprehensive Specialized Hospital from February to June 2019.

| **Vaginal infections** | **Frequency** | **Percent** |
| --- | --- | --- |
| Bacterial vaginosis (BV) | 52 | 12.3 |
| Aerobic vaginitis (AV) | 6 | 1.4 |
| Candidiasis | 19 | 4.5 |
| Trichomoniasis | 5 | 1.2 |
| Total | 82 | 19.2 |
| **Mixed infection** |  | |
| BV + AV | 23 | 5.5 |
| BV + candidiasis | 7 | 1.7 |
| BV + AV + trichomoniasis | 3 | 0.7 |
| AV + candidiasis | 1 | 0.2 |
| AV + trichomoniasis | 1 | 0.2 |
| Total | 35 | 8.3 |
| Normal women without any vaginal infections | 305 | 72.3 |
| Women with normal scores for BV and AV | 326 | 77.3 |
| Total | 422 | 100 |
