# Supplementary material for: Prevalence of bacterial vaginosis and aerobic vaginitis and their associated risk factors among pregnant women from northern Ethiopia: A cross-sectional study
Source: PLoS One. 2022 Feb 25;17(2):e0262692. doi: 10.1371/journal.pone.0262692 (PMC8880645; doi:10.1371/journal.pone.0262692)
Supplement: S3 Table — (DOCX) [file pone.0262692.s004.docx]

**Supplementary Information**

Table 3. Univariate and multivariate analysis of factors associated with bacterial vaginosis among pregnant women attending antenatal care in Ayder Comprehensive Specialized Hospital from February to June 2019.

| **Variables** | | **BV positive**  **n (%)** | **BV negative**  **n (%)** | **Univariate** | | **Multivariate** | |
| --- | --- | --- | --- | --- | --- | --- | --- |
|  |  |  |  | **COR (95% CI)** | ***P*-value** | **AOR (95% CI)** | ***P*-value** |
| Age(year) | ≤ 20 | 8 (21.6) | 29 (78.4) | 0.810 (0.324, 2.021) | 0.651 |  |  |
|  | 21-29 | 56 (20.7) | 214 (79.3) | 0.854 (0.489,1.490) | 0.578 |  |  |
|  | ≥ 30 | 21 (18.3) | 94 (81.7) | 1 |  |  |  |
| Place of residence | Urban | 80 (20.0) | 320 (80.0) | 1.176 (0.421, 3.285) | 0.756 |  |  |
|  | Rural | 5 (22.7) | 17 (77.3) | 1 |  |  |  |
| Educational status | Unable to read and write | 4 (21.0) | 15(79.0) | 0.645 (0.196, 2.123) | 0.470 | 0.734 (0.207, 2.605) | 0.632 |
|  | Primary | 17 (20.7) | 65 (79.3) | 0.657 (0.326, 1.323) | 0.240 | 0.866 (0.389, 1.930) | 0.725 |
|  | Secondary | 42 (24.6) | 129 (75.4) | 0.528 (0.298, 0.934) | 0.028^*^ | 0.645 (0.338, 1.228) | 0.182 |
|  | College and above | 22 (14.7) | 128 (85.3) | 1 |  |  |  |
| Occupation | Employee | 16 (14.4) | 95 (85.6) | 1.979 (0.967, 4.052) | 0.062 | 1.445 (0.650, 3.214) | 0.366 |
|  | Housewife | 47 (21.1) | 176 (78.9) | 1.248 (0.699, 2.229) | 0.454 | 1.081(0.579, 2.020) | 0.619 |
|  | Others | 22 (25.0) | 66 (75.0) | 1 |  |  |  |
| Marital status | Unmarried | 5 (33.3) | 10 (66.7) | 1 |  |  |  |
|  | Married | 78 (19.4) | 324 (80.6) | 2.077 (0.690, 6.249) | 0.193 | 1.557 (0.470. 5.155) | 0.469 |
|  | Divorced/widowed | 2 (25.0) | 3 (75.0) | 0.750 (0.093, 6.043) | 0.787 | 2.971 (0.404. 21.838) | 0.285 |
| Cigarette smoking | Yes | 0 (0.0) | 1 (100.0) | - | - |  |  |
|  | No | 85 (20.2) | 336 (79.8) | 1 |  |  |  |
| HIV | Positive | 3 (25.0) | 9 (75.0) | 0.750 (0.199, 2.833) | 0.671 |  |  |
|  | Negative | 82 (20.0) | 328 (80.0) | 1 |  |  |  |
| Syphilis | Positive | 1 (33.3) | 2 (66.7) | 0.501 (0.045, 5.597) | 0.575 |  |  |
|  | Negative | 84 (20.0) | 335 (80.0) | 1 |  |  |  |
| Condom use | Yes | 6 (19.4) | 25 (80.6) | 1.055 (0.419, 2.659) | 0.910 |  |  |
|  | No | 79 (20.2) | 312 (79.8) | 1 |  |  |  |
| Previous fungal infection | Yes | 8 (24.2) | 25 (75.8) | 0.771 (0.335, 1.776) | 0.542 |  |  |
|  | No | 77 (19.8) | 312 (80.2) | 1 |  |  |  |
| Number of LTSP | One | 72 (19.8) | 291 (80.2) | 1 |  |  |  |
|  | Two and above | 13 (22.0) | 46 (78.0) | 0.875 (0.449, 1.707) | 0.696 |  |  |
| Number of pantyliner used/day | 1-2/day | 63 (21.1) | 235 (78.9) | 1 |  |  |  |
|  | 1/2-4 days | 22 (17.7) | 102 (82.3) | 1.243 (0.711, 2.089) | 0.428 |  |  |
| Douching using water | Once daily | 27 (22.7) | 92 (77.3) | 1 |  |  |  |
|  | More than once daily | 58 (19.1) | 245 (80.9) | 1.240 (0.740, 2.076) | 0.414 |  |  |
| Douching using soap | Yes | 8 (18.6) | 35 (81.4) | 1.115 (0.497, 2.502) | 0.791 |  |  |
|  | No | 77 (20.3) | 302 (79.7) | 1 |  |  |  |
| Previous BV/GTI | Yes | 11 (17.7) | 51 (82.3) | 1.200 (0.596, 2.416) | 0.610 |  |  |
|  | No | 74 (20.6) | 286 (79.4) | 1 |  |  |  |
| Previous history of abortion | Once | 15 (23.4) | 49 (76.6) | 0.783 (0.412, 1.485) | 0.453 |  |  |
|  | Spontaneously | 7 (21.9) | 25 (78.1) | 0.856 (0.354, 2.067) | 0.729 |  |  |
|  | No | 63 (19.3) | 263 (80.7) | 1 |  |  |  |
| Number of the sexual partner in the last 12 months | One | 85 (20.2) | 336 (79.8) | 1 |  |  |  |
|  | More than two | 0(0) | 1(100.0) | - | - |  |  |
| Gestational age | 1^st^ trimester | 9 (17.6) | 42 (82.4) | 0.920 (0.401, 2.109) | 0.843 | 0.843 (0.359, 1.980) | 0.695 |
|  | 2^nd^ trimester | 49 (23.7) | 158 (76.3) | 0.635 (0.377, 1.072) | 0.089 | 0.563 (0.324, 0.979) | 0.042^*^ |
|  | 3^rd^ trimester | 27 (16.5) | 137 (83.5) | 1 |  |  |  |
| Number of pregnancy | Primigravida | 28 (17.7) | 130 (82.3) | 1 |  |  |  |
|  | Multigravida | 57 (21.6) | 207 (78.4) | 1.278 (0.773, 2.114) | 0.334 |  |  |

COR=Crude odd ratio, AOR=Adjusted odd ratio, CI =confidence interval, ANC = Antenatal care, HIV = Human immunodeficiency virus, LTSP = Lifetime sexual partner, BV = Bacterial vaginosis, GTI = Genital Tract Infection

* = Significant association
