# Supplementary material for: Prevalence of bacterial vaginosis and aerobic vaginitis and their associated risk factors among pregnant women from northern Ethiopia: A cross-sectional study
Source: PLoS One. 2022 Feb 25;17(2):e0262692. doi: 10.1371/journal.pone.0262692 (PMC8880645; doi:10.1371/journal.pone.0262692)
Supplement: S4 Table — (DOCX) [file pone.0262692.s005.docx]

**Supplementary Information**

Table 4. Univariate and multivariate analysis of factors associated with aerobic vaginitis among pregnant women attending antenatal care in Ayder Comprehensive Specialized Hospital from February to June 2019.

| **Variables** | | **AV positive**  **n (%)** | **AV negative**  **n (%)** | **Univariate analysis** | | **Multivariate analysis** | |
| --- | --- | --- | --- | --- | --- | --- | --- |
|  |  |  |  | **COR (95% CI)** | ***P*-value** | **AOR (95% CI)** | ***P*-value** |
| Age (year) | ≤ 20 | 3 (8.1) | 34 (91.9) | 1 |  |  |  |
|  | 21-29 | 20 (7.4) | 250 (92.6) | 1.103 (0.311, 3.909) | 0.879 |  |  |
|  | ≥ 30 | 11 (9.6) | 104 (91.4) | 0.834 (0.220, 3.167) | 0.790 |  |  |
| Residence | Urban | 34 (8.5) | 366 (91.5) | 1 | 0.240 |  |  |
|  | Rural | 0 (0) | 22 (100) | - |  |  |  |
| Educational status | Unable to read and write | 1 (5.3) | 18 (94.7) | 0.750 (0.085, 6.588) | 0.795 | 0.671(0.064, 7.038) | 0.739 |
|  | Primary | 6 (7.3) | 76 (92.7) | 0.528 (0.165, 1.692) | 0.282 | 0.499 (0.133, 1.873) | 0.303 |
|  | Secondary | 21 (12.3) | 150 (87.7) | 0.298 (0.117, .759) | 0.011^*^ | 0.292 (0.102, 0.833) | 0.021^*^ |
|  | College and above | 6 (4.0) | 144 (96.0) | 1 |  |  |  |
| Occupation | Employee | 6 (5.4) | 105 (94.6) | 3.311(1.216, 9.014) | 0.019^*^ | 2.003 (0.654, 6.141) | 0.224 |
|  | Housewife | 14 (6.3) | 209 (93.7) | 2.824 (1.286, 6.203) | 0.010^*^ | 2.856 (1.250, 6.523) | 0.013^*^ |
|  | Others | 14 (15.9) | 74 (84.1) | 1 |  |  |  |
| Marital status | Unmarried | 3 (20.0) | 12 (80.0) | 1 |  |  |  |
|  | Married | 30 (7.5) | 372 (92.5) | 3.100 (.829, 11.590) | 0.093 | 3.182 (.771. 13.121) | 0.109 |
|  | Divorced/widowed | 1 (20.0) | 4 (80.0) | 1.000 (.080, 12.557) | 1.000 | 1.998 (.128, 31.175) | 0.622 |
| Cigarette smoking | Yes | 0 (0) | 1 (100.0) | - |  |  |  |
|  | No | 34 (8.1) | 387 (91.9) | 1 |  |  |  |
| HIV | Positive | 2 (16.7) | 10 (83.3) | 0.423 (0.089, 2.015) | 0.280 |  |  |
|  | Negative | 32 (7.8) | 378 (92.2) | 1 |  |  |  |
| Syphilis | Positive | 0 (0) | 3 (100.0) | - | - |  |  |
|  | Negative | 34 (8.1) | 385 (91.9) | 1 |  |  |  |
| Condom use | Yes | 3 (9.7) | 28 (90.3) | 1 |  |  |  |
|  | No | 31 (7.9) | 360 (92.1) | 1.244 (0.358, 4.325) | 0.731 |  |  |
| Previous fungal infection | Yes | 5 (15.2) | 28 (84.8) | 0.451(0.162, 1.256) | 0.128 | 0.444(0.152, 1.294) | 0.1377 |
|  | No | 29 (7.5) | 360 (92.5) | 1 |  |  |  |
| Number of LTSP | One | 28 (7.7) | 335 (92.3) | 1 |  |  |  |
|  | Two and above | 6 (10.2) | 53 (89.8) | 0.738 (0.292, 1.868) | 0.522 |  |  |
| Number of pantyliner used/day | 1-2/day | 26 (8.7) | 272 (91.3) | 1 |  |  |  |
|  | 1/2-4 days | 8 (6.5) | 116 (93.5) | 1.386 (0.609, 3.152) | 0.436 |  |  |
| Douching using water | Once daily | 7(5.9) | 112 (94.1) | 1 |  |  |  |
|  | More than once daily | 27(8.9) | 276 (91.1) | 0.639 (0.270, 1.510) | 0.307 |  |  |
| Douching using soap | Yes | 2 (4.7) | 41 (95.3) | 1.890 (0.437, 8.179) | 0.394 |  |  |
|  | No | 32 (8.4) | 347 (91.6) | 1 |  |  |  |
| Previous BV/GTI | Yes | 4 (6.5) | 58 (93.5) | 1.318 (0.448, 3.881) | 0.616 |  |  |
|  | No | 30 (8.3) | 330 (91.7) | 1 |  |  |  |
| Previous history of abortion | Once | 6 (8.4) | 58 (91.6) | 0.768 (.301, 1.962) | 0.582 |  |  |
|  | Spontaneously | 4 (12.5) | 28 (87.5) | 0.556 (0.180, 1.717) | 0.308 |  |  |
|  | No | 24 (7.4) | 302 (92.6) | 1 |  |  |  |
| Number of the sexual partner in the last 12 months | One | 34 (8.1) | 387 (91.9) | 1 |  |  |  |
|  | More than two | 0 (0) | 1 (100.0) | - |  |  |  |
| Gestational age | 1^st^ trimester | 4 (7.8) | 47 (92.2) | 0.928 (0.286, 3.013) | 0.901 |  |  |
|  | 2^nd^ trimester | 18 (8.7) | 189 (91.3) | 0.829 (0.387, 1.774) | 0.629 |  |  |
|  | 3^rd^ trimester | 12 (7.3) | 152 (92.7) | 1 |  |  |  |
| Number of pregnancy | Primigravida | 11 (7.0)) | 147 (93.0) | 1 |  |  |  |
|  | Multigravida | 23 (8.7) | 241 (91.3) | 0.784 (0.371, 1.655) | 0.523 |  |  |

COR=Crude odds ratio, AOR=Adjusted odds ratio, CI=confidence interval, ANC = Antenatal care, HIV = Human immunodeficiency virus, LTSP= Lifetime sexual partner, BV= Bacterial vaginosis, GTI= Genital tract infection, AV = Aerobic vaginitis

* = Significant association
