# Supplementary material for: Prevalence of bacterial vaginosis and aerobic vaginitis and their associated risk factors among pregnant women from northern Ethiopia: A cross-sectional study
Source: PLoS One. 2022 Feb 25;17(2):e0262692. doi: 10.1371/journal.pone.0262692 (PMC8880645; doi:10.1371/journal.pone.0262692)
Supplement: S5 Table — (DOCX) [file pone.0262692.s006.docx]

**Supplementary Information**

Table 5. Percentage of antibacterial susceptibility pattern of all *Staphylococcus* isolates (n=30) from Ayder Comprehensive Specialized Hospital from February to June 2019.

| ***Staphylococcus* strains (n)** | **Pattern** | **Antibacterial drugs** | | | | | | | | |
| --- | --- | --- | --- | --- | --- | --- | --- | --- | --- | --- |
|  |  | **PEN (%)** | **CIP (%)** | **DA (%)** | **E (%)** | **CN (%)** | **TE (%)** | **CAF (%)** | **DOX (%)** | **SXT (%)** |
| CoNS  (17) | S | 0 (0.0) | 14 (82.4) | 13 (76.6) | 4 (23.6) | 15 (88.2) | 7 (41.2) | 13 (76.5) | 11 (64.7) | 7 (41.2) |
|  | I | 0 (0.0) | 0 (0.0) | 2 (11.7) | 2 (11.8) | 1 (5.9) | 5 (29.4) | 1 (5.9) | 2 (11.8) | 1 (5.9) |
|  | R | 17 (100) | 3 (17.6) | 2 (11.7) | 11 (64.6) | 1 (5.9) | 5 (29.4) | 3 (17.6) | 4 (23.5) | 9 (52.9) |
| *S. aureus*  (13) | S | 0 (0.0) | 12 (92.3) | 9 (69.2) | 0 (0.0) | 10 (76.9) | 6 (46.2) | 11 (84.6) | 8 (61.5) | 6 (46.2) |
|  | I | 0 (0.0) | 1 (7.7) | 2 (15.4) | 6 (46.2) | 1 (7.7) | 3 (23.0) | 0 (0.0) | 2 (15.4) | 0 (0.0) |
|  | R | 13 (100) | 0 (0.0) | 2 (15.4) | 7 (53.8) | 2 (15.4) | 4 (30.8) | 2 (15.4) | 3 (23.1) | 7 (53.8) |
| Total  (30) | S | 0 (0.0) | 26 (86.7) | 22 (73.4) | 4 (13.3) | 25 (83.3) | 13 (43.3) | 24 (80.0) | 19 (63.4) | 13 (43.3) |
|  | I | 0 (0.0) | 1 (3.3) | 4 (13.3) | 8 (26.6) | 2 (6.7) | 8 (26.7) | 1 (3.3) | 4 (13.3) | 1 (3.3) |
|  | R | 30 (100.0) | 3 (10.0) | 4 (13.3) | 18 (60.0) | 3 (10.0) | 9 (30.0) | 5 (16.7) | 7 (23.3) | 16 (53.4) |
