# Supplementary material for: Prevalence of bacterial vaginosis and aerobic vaginitis and their associated risk factors among pregnant women from northern Ethiopia: A cross-sectional study
Source: PLoS One. 2022 Feb 25;17(2):e0262692. doi: 10.1371/journal.pone.0262692 (PMC8880645; doi:10.1371/journal.pone.0262692)
Supplement: S6 Table — (DOCX) [file pone.0262692.s007.docx]

**Supplementary Information**

Table 6. Percentage of antibacterial susceptibility pattern of all *Enterobacteriaceae* isolates (n=14) from the Ayder Comprehensive Specialized Hospital from February to June 2019.

| ***Enterobacteriaceae* (n)** | **Pattern** | **Antibacterial drugs** | | | | | | | | | | |
| --- | --- | --- | --- | --- | --- | --- | --- | --- | --- | --- | --- | --- |
|  |  | **MER (%)** | **CIP (%)** | **TOB (%)** | **AK (%)** | **AMC (%)** | **CN (%)** | **TE (%)** | **CAF (%)** | **AM (%)** | **DOX (%)** | **SXT (%)** |
| ***E. coli* (11)** | S | 10 (90.9) | 11 (100.0) | 6 (54.5) | 6 (54.5) | 0 (0.0) | 10 (90.9) | 7 (63.6) | 11 (100.0) | 0 (0.0) | 7 (63.6) | 11 (100.0) |
|  | I | 1 (9.1) | 0 (0.0) | 2 (18.2) | 1 (9.1) | 0 (0.0) | 1(9.1) | 2 (18.2) | 0 (0.0) | 1 (9.1) | 2 (18.2) | 0 (0.0) |
|  | R | 0 (0.0) | 0 (0.0) | 3 (27.3) | 4 (36.4) | 11 (100) | 0 (0.0) | 2 (18.2) | 0(0.0) | 10 (90.1) | 2 (18.2) | 0 (0.0) |
| ***Citrobacter* spp. (2)** | S | 1(50.0) | 2 (100.0) | 0 (0.0) | 2 (100) | 0 (0.0) | 2 (100.0) | 1 (50.0) | 2 (100.0) | 0 (0.0) | 0 (0.0) | 0 (0.0) |
|  | I | 1(50.0) | 0 (0.0) | 0 (0.0) | 0 (0.0) | 0 (0.0) | 0 (0.0) | 0 (0.0) | 0 (0.0) | 0 (0.0) | 0 (0.0) | 0 (0.0) |
|  | R | 0 (0.0) | 0 (0.0) | 2 (100) | 0 (0.0) | 2 (100.0) | 0 (0.0) | 1 (50.0) | 0 (0.0) | 2 (100) | 2 (100.0) | 2 (100.0) |
| ***K. pneumoniae* (1)** | S | 1 (100.0) | 1 (100.0) | 0 (0.0) | 1 (100.0) | 0 (0.0) | 1 (100.0) | 1 (100) | 1 (100) | 0 (0.0) | 1 (100.0) | 0 (0.0) |
|  | I | 0 (0.0) | 0 (0.0) | 0 (0.0) | 0 (0.0) | 0 (0.0) | 0 (0.0) | 0 (0.0) | 0 (0.0) | 0 (0.0) | 0 (0.0) | 1 (100.0) |
|  | R | 0 (0.0) | 0 (0.0) | 1(100.0) | 0 (0.0) | 1 (100) | 0 (0.0) | 0 (0.0) | 0 (0.0) | 1 (100.0) | 0 (0.0) | 0 (0.0) |
| **Total (14)** | S | 12 (85.6) | 14 (100.0) | 6 (42.8) | 9(64.3) | 0 (0.0) | 13 (92.9) | 9 (64.3) | 14 (100.0) | 0 (0.0) | 8 (57.1) | 11 (78.5) |
|  | I | 2 (14.4) | 0 (0.0) | 2 (14.4) | 1 (7.1) | 0 (0.0) | 1 (7.1) | 2 (14.3) | 0 (0.0) | 1 (7.1) | 2 (14.3) | 1 (7.1) |
|  | R | 0 (0.0) | 0 (0.0) | 6 (42.8) | 4 (28.6) | 14 (100.0) | 0 (0.0) | 3 (21.4) | 0 (0.0) | 13 (92.9) | 4 (28.6) | 2 (14.4) |

MER = Meropenem, CIP = Ciprofloxacin, TOB = Tobramycin, AK = Amikacin, AMC = Amoxicillin/clavulanate, CN = Gentamycin, TE = Tetracycline, CAF = Chloramphenicol, AM = Ampicillin, DOX = Doxycycline, SXT = Trimethoprim/sulfamethoxazole, S = Sensitive, I = Intermediate, R = Resistant
