# Supplementary material for: Prevalence of bacterial vaginosis and aerobic vaginitis and their associated risk factors among pregnant women from northern Ethiopia: A cross-sectional study
Source: PLoS One. 2022 Feb 25;17(2):e0262692. doi: 10.1371/journal.pone.0262692 (PMC8880645; doi:10.1371/journal.pone.0262692)
Supplement: S7 Table — (DOCX) [file pone.0262692.s008.docx]

**Supplementary Information**

Table 7. Percentage of multiple drug resistance pattern of all bacterial isolates (n=44) from Ayder Comprehensive Specialized Hospital from February to June 2019.

| Bacterial isolates | Total  n (%) | Antimicrobial resistance pattern | | | | | | |
| --- | --- | --- | --- | --- | --- | --- | --- | --- |
|  |  | **R0** | **R1** | **R2** | **R3** | **R4** | $\boldsymbol{\geq}$ **R5** | **Multiple drug resistance** |
| *Enterobacteriaceae* | 14 (31.8) | 0 (0.0) | 0 (0.0) | 5 (35.7) | 5 (35.7) | 1 (7.2) | 3 (21.4) | 8 (57.1) |
| *E. coli* | 11 (25.0) | 0 (0.0) | 0 (0.0) | 5 (45.4) | 4 (36.4) | 1 (9.1) | 1 (9.1) | 5 (45.5) |
| *Citrobacter* spp. | 2 (4.5) | 0 (0.0) | 0 (0.0) | 0 (0.0) | 0 (0.0) | 0 (0.0) | 2 (100.0) | 2 (100.0) |
| *K. pneumoniae* | 1 (2.3) | 0 (0.0) | 0 (0.0) | 0 (0.0) | 1(100.0) | 0 (0.0) | 0 (0.0) | 1 (100.0) |
| *Staphylococcus* strains | 30 (68.2) | 0 (0.0) | 7 (23.3) | 9 (30.0) | 4 (13.3) | 2 (6.7) | 8 (26.7) | 12 (40.0) |
| CoNS | 17 (38.6) | 0 (0.0) | 4 (23.5) | 5 (29.4) | 2 (11.8) | 2 (11.8) | 4 (23.5) | 8 (62.5) |
| *S. aureus* | 13 (29.6) | 0 (0.0) | 3 (23.0) | 4 (30.8) | 2 (15.4) | 0 (0.0) | 4 (30.8) | 4 (30.8) |
| Total | 44(100.0) | 0 (0.0) | 7 (15.9) | 14 (31.8) | 9 (20.5) | 3 (6.8) | 11 (25.0) | 20 (45.5) |

CoNS = Coagulase negative staphylococci, *S. aureus* = *Staphylococcus aureus*, *E. coli* = *Escherichia coli*, *K. pneumoniae* = *Klebsiella pneumonia*

R0: susceptible to all antibiotic, R1: resistant to 1 antibiotic, R2: resistant to 2 antibiotics, R3: resistant to 3 antibiotics, R4: resistant to 4 antibiotics, ≥ R5: resistant to 5 or more antibiotics
