# Supplementary material for: Prevalence of bacterial vaginosis and aerobic vaginitis and their associated risk factors among pregnant women from northern Ethiopia: A cross-sectional study
Source: PLoS One. 2022 Feb 25;17(2):e0262692. doi: 10.1371/journal.pone.0262692 (PMC8880645; doi:10.1371/journal.pone.0262692)
Supplement: Attachment — Submitted filename: Response to Reviewers.docx [file pone.0262692.s010.docx]

Dear Dr. Caroline Mitchell

Academic Editor of PLOS ONE

Subject : Submission of revised manuscript entitled "**Prevalence of bacterial vaginosis and aerobic vaginitis and their associated risk factors among pregnant women from northern Ethiopia: a cross-sectional study**" (ID: PONE-D-21-28305R1).

Thank you for your email of 3 December, 2021 enclosing the editor and reviewers ̓comments.We also greatly appreciate the reviewers for their complimentary comments and suggestions. We have carefully reviewed the comments and have revised the manuscript accordingly. Our point to point responses are given below. (The reviewer’s comments are in italics). Changes to the manuscript are indicated in yellow highlighted sentences/words. We hope that you find our responses satisfactory and that the manuscript is now acceptable for publication. **Anyway, we should be grateful if you let us know about our further changes required.**

Yours sincerely,

**Corresponding author**: Morteza Saki

Mailing address: Department of Microbiology, Faculty of Medicine, Ahvaz Jundishapur University of Medical Sciences, Ahvaz, Iran

P.O. Box: 159

Postal Code: 61357-15794

Email: mortezasaki1981@gmail.com

The followings are point-by-point responses :

*The abstract states: “BV was significantly associated with symptoms of white homogeneous vaginal discharge (P < 0.001) and second trimester (P = 0.042). However, AV was significantly associated with secondary school (P = 0.021) and housewife status (P = 0.013).” It would be helpful to include the reference category, i.e: BV was more common in symptomatic vs. asymptomatic people, and in second trimester vs. first trimester samples.*

**Response (R): We fixed what you requested but due to limitation of the word count of abstract, we have to delete some part of manuscript accordingly to reach 300 words.**

*Line 102-103 state: “…numerous research have looked at the link between AV and pregnancy outcomes..” As noted previously, I think this is a mischaracterization. There are very few studies, actually, especially when compared to BV.*

**Response (R): Fixed.**

*Line 115 states that groups have a higher predisposition for BV “…because they have fewer lactobacilli that produce hydrogen peroxide.” I would delete this clause because the causal link between the described features and lower lactobacilli and BV is not proven. It is all associations.*

**Response (R): Fixed.**

*Line 311-314: states “…even higher prevalence was reported among pregnant women who douche their vagina frequently using water (8.9%), those who didn’t use soap during douching (8.4%)”  Both clauses need to describe to what they are compared – the table implies that everyone douched at least once daily – is this true? How is douching characterized?  The second clause also implies that everyone is douching – it would be better represented by use of soap within those who douche. If what is meant is vaginal cleansing with fingers or a cloth, this should be specified. Douching means irrigating the vagina with a liquid.*

**Response (R): Fixed.**

*Line 515-524: The limitations paragraph should go before the Conclusion section. Additionally, please break out the list of limitations into a more readable set of sentences.*

**Response (R): Fixed.**

*Minor typographical/grammatical errors:*

*Line 90-91: “However, both AV and BV are vaginal dysbiosis that characterized by the lactobacilli reduction”  - dysbiosis needs to be plural and either the “that” removed, or an “are” added after it. Also, “the lactobacilli reduction” should be “the reduction in lactobacilli.”*

**Response (R): Fixed.**

*Line 97: lactobacillus should be italicized and capitalized*

**Response (R): Fixed.**

*Line 99: “These enzymes sialic stimulate the”. Please remove the word “sialic” here*

**Response (R): Fixed.**

*Line 188: “…Lactobacillus morphotypes i.e. that no morphotypes = 4.” Please remove the word “that”*

**Response (R): Fixed.**

*Line 233-234 states “…study participants were secondary school…”. Do you mean that participants had completed secondary school? Or had some secondary school education? Please specify.*

**Response (R): Fixed.**

*Line 255 states “…results were not significantly associated (P = 0.549).” – please add what results were not associated with (I think it is symptoms, but to make it unambiguous this should be specified).*

**Response (R): Fixed.**

*Lines 285-287: “However, pregnant women with the second trimester had a higher prevalence of BV (23.7%) than those who were in the first and third trimester with a prevalence of (17.6%) and (16.5%), respectively. Pregnant women in the second trimester, had significantly higher BV (P = 0.042).” – In the first sentence, it should be be “…pregnant women sampled in the second…”. In the second sentence, please specify higher in comparison to…(I believe it is those sampled in the third trimester, but again, this should be explicitly stated). (Same comments for line 314-317, however, here you state that the people in second trimester had higher prevalence than those in both first and third trimester, but it appears that the reference group is third trimester only).(Also line 413-414)*

**Response (R): Fixed.**

*Line 304 “…than 21-29 years and those lower than 20 years age…” Please include “women aged” after than, and change the latter part to “20 years of age”*

**Response (R): Fixed.**

*Line 311: pantyliner should be plural*

**Response (R): Fixed.**

*Line 316-317: “…who were got pregnant for the first time…” Please remove the word got*

**Response (R): Fixed.**

*Line 318: “…who were pregnant many times” Please change to “who had been pregnant before…”*

**Response (R): Fixed.**

*Line 318 states that “…but they were not significantly associated…” – to what does “they” refer? Do you mean that gravidity was not associated with AV? If so, please state that.*

**Response (R): Fixed.**

*Line 320: “However, after adjustment of confounders in multivariate analyses, secondary school [0.292 (0.102, 0.833)], P = 0.021] and housewife [2.856 (1.250, 6.523), P = 0.013] pregnant women remained independently associated with a decreased (preventive) and increased likelihood of AV positive, respectively. Please specify reference categories.*

**Response (R): Fixed.**

*Line 333-334 states: “different Staphylococcus strains and Enterobacteriaceae species,” – I believe you mean species for both. A strain is a specific isolate of a given species, and the next sentence talks about different Staph species.  I do think that the term strains is used appropriately in Table 5.*

**Response (R): Fixed.**

*Line 395 states “…the uncultivated bacterial vaginosis-associated bacterium.” This should be “……the uncultivated bacterial vaginosis-associated bacterium-1 (BVAB1).”  (there are three: BVAB1, BVAB2 and BVAB3, which is now known as Mageebacillus indolicus, which is why it is important to specify).*

**Response (R): Fixed.**

*Line 419: “In contrast to the current study, a study reported from South Africa, revealed that HIV-positive item was significantly associated with BV”. Please change the “that HIV-positive status…”*

**Response (R): Fixed.**

*Line 434” “…knowledge, the AV and its associated”. Please delete “the”*

**Response (R): Fixed.**

*Line 436-438: “In this study, no one of socio-demographic and sexual and behavioral characteristics variables was significantly associated with the prevalence of AV in pregnant women except for education and occupation status that was in contrast…” Please delete “one of” and change “was” to “were”*

**Response (R): Fixed.**

*Line 455: Please add “A” at the beginning of this sentence: “Previous study from China reported a higher prevalence rate of mixed AV cases with other infections…”*

**Response (R): Fixed.**

*Line 476: “Also, the gentamicin with susceptibility rate of 92.9% was the third effective antibiotic against Enterobacteriaceae.” Please change to “Also, gentamicin with a susceptibility rate of 92.9% was the third most effective…”*

**Response (R): Fixed.**

*Line 506-512: “The prevalence of BV was higher among symptomatic (35.1%) than asymptomatic pregnant women (16.8%). White vaginal discharge and second trimester were the risk factor and protective item for BV, respectively. Whereas, secondary school and housewife were also found protective and risk factor for AV, respectively. The Staphylococcus spp. were more prevalent than the Enterobacteriaceae in AV pregnant women. Amoxicillin/clavulanate had the highest overall resistance rate against Enterobacteriaceae, while the overall resistance rate of Staphylococcus spp.was observed against penicillin.” – This simply restates what has already been discussed and could be deleted.*

**Response (R): Thank you for your suggestion. However, Due to the high lenghth of the manuscript, we prefer to remain this summery of the study.**

*Reviewer #1:*

*Just some examples of minor errors in the revised manuscript with track changes:
Line 446: "In line with the current study, Hassan et al. [11] from Egypt, showed that..."
Line 451: "In line with the current study, Salinas et al. [50] and Pacha-Herrera et al. [76] from Ecuador, did"*

**Response (R): Fixed.**
